# Effects of intravenous diltiazem in a rat model of experimental coronary thrombotic microembolism

**DOI:** 10.3892/etm.2013.1263

**Published:** 2013-08-16

**Authors:** YUPENG BAI, LIQUN HU, JIE WU, YE GU, LUN LI, BO GAO, HONG JIANG

**Affiliations:** 1 Department of Cardiology, Renmin Hospital of Wuhan University, Wuhan 430060;; 2 Department of Cardiology, Puai Hospital, Huazhong University of Science and Technology, Wuhan 430030, P.R. China

**Keywords:** coronary thrombosis, microembolism, no flow phenomenon, diltiazem

## Abstract

The aim of this study was to assess the feasibility of evaluating the therapeutic effects of intravenous diltiazem in a newly established rat model of coronary thrombotic micro-embolism (CME). CME was induced by injecting 0.199 ml saline containing 5 mg of automicrothrombotic particulates (∼10 *μ*m) into the aorta of Sprague Dawley rats. The injection was carried out over 10 sec using a tuberculin syringe with a 28-gauge needle. The CME model rats were randomly divided into untreated (CME, n=38) and diltiazem-treated (CME+DIL, n=38) groups. Diltiazem (1 mg/ml, 50 *μ*g/min/kg) was intravenously injected using an infusion pump through the tail vein for 175 min, 5 min following the injection of the automicrothrombotic particulates. Hemodynamic measurements, echocardiography and pathohistological examinations were performed at various time-points (3 h, 24 h and 7 and 28 days) postoperatively. Arteriolar thrombosis, multifocal myocardial necrosis, inflammatory cell infiltration with markedly increased myocardial tumor necrosis factor α (TNF-α) and interleukin-6 (IL-6) expression, reduced left ventricular (LV) systolic function and increased plasma von Willebrand factor (vWF), endothelin-1 (ET-1) and serum c-troponin I (c-TnI) levels (indicating vascular endothelial injury and myocardial necrosis) were observed in the CME model rats. These pathological responses in CME rats were partly attenuated by intravenous diltiazem treatment. The present CME model is suitable for evaluating the therapeutic effects of intravenous diltiazem; intravenous diltiazem treatment significantly improved cardiac function through alleviating inflammatory responses and microvascular thrombotic injury in this rat model of CME.

## Introduction

Coronary thrombotic microembolism (CME) may be induced by spontaneous plaque rupture and disrupted plaque components produced during coronary interventions. CME has been shown to lead to myocardial microinfarcts, as reflected by elevated creatine kinase (CK) and troponin I (TnI) (1–7), which may partly contribute to impaired microvascular perfusion and result in the ‘slow’ or ‘no-reflow’ phenomenon (6–10). A number of efforts, including intravascular thrombolysis drug application and thrombosis suction, have been attempted for the prevention and treatment of CME and CME-induced myocardial impairment (11–16). Previous studies have demonstrated that calcium antagonists are capable of relieving microvascular spasm (17,18) and that the intravascular application of diltiazem may attenuate coronary artery spasms in patients with microvascular angina or acute myocardial infarction with ‘no-reflow’ phenomenon (19,20). In our previous experimental study, a rat CME model was established by the aortic injection of automicrothrombotic particulates (9). In the present study, we examined whether this model is suitable for testing and reflecting the therapeutic effects of drugs that may be capable of attenuating coronary microembolization. It was hypothesized that intravenous diltiazem, an agent with proven clinical effectiveness in reducing coronary microembolization, may attenuate the cardiac dysfunction and pathological changes in the rat CME model.

## Materials and methods

### 

#### Animals

A total of 152 adult male Sprague Dawley rats (weight, 250–350 g; age, 12 weeks) were used in the present study. Thirty-two rats were used for the dose-finding pilot study. Forty rats were subjected to an aortic saline injection (sham group, n=40) and 80 rats underwent procedures to induce CME, as described previously (9). Briefly, blood (0.5 ml) was obtained from the ventral tail artery of all rats 1 day prior to surgery (21). The blood was left to clot for 1 h and then dried overnight at 37°C (9,22). The dried blood was fragmented into thrombotic particulates with a Sabi Crush Easy Pill Smasher (SABI Co., Palo Alto, CA, USA) for 5 min. Subsequently, 5 mg thrombotic particulates were dissolved in 0.2 ml saline and filtered through a 45-*μ*m metal filter. The filtrates were collected and the mixed solution (1 *μ*l) was utilized for a particulate number count and size determination using a TS-M2 micrometer (OPLENIC Manufacturer, HangZhou, China). Examination revealed that the number of particulates with a diameter of 10–45 *μ*m was ∼600,000, whereas the majority of particulates (>90%, ∼16,000,000) had a diameter <10 *μ*m. The remaining 0.199 ml filtrates were injected into sodium pentobarbital-anesthetized (50 mg/kg, i.p.) rats. The second intercostal space was exposed through parasternotomy and a microretractor was used to separate the second and third rib in order to adequately expose the operating region under strictly aseptic conditions. Following the removal of the pericardium, the ascending aorta was visualized. The ascending aorta was temporarily clamped by a microvascular clip and 0.199 ml filtrate or 0.2 ml saline was injected into the aorta over 10 sec using a 28-gauge tuberculin syringe. The mean quantity of hemorrhage ranged from 0.5 to 1.0 ml following injection into animals without massive hemorrhage. During surgery, the animals were monitored with electrocardiograms using standard lead II through subcutaneous needle electrodes. Penicillin (800,000 IU, i.p.) was administered daily for 7 days to animals studied at 7 and 28 days after the aortic injection of automicrothrombotic particulates or saline. Four rats died immediately after the injection of automicrothrombotic particulates (three due to large hemorrhage and one due to malignant arrhythmia). The CME model rats that survived were randomly assigned to the untreated (CME group, n=38) or diltiazem-treated CME groups (CME+DIL group, n=38). In the latter group, diltiazem (1 mg/ml) was intravenously injected at an infusion rate of 50 *μ*g/min/kg using an infusion pump (Perfusor®, TCI-II; Guangxi Veryark Technology Co., Ltd., Guangxi, China) through the tail vein for 175 min, 5 min following the injection of automicrothrombotic particulates. The dose of drug used in the main study was selected based on the results of the pilot study (as described in Results). The rats were examined and sacrificed at 3 h, 24 h, 7 days and 28 days postoperatively (n=8–10 at each time point).

Experiments were approved by the Tongji Medical College Council on the Animal Care Committee of Huazhong University of Science and Technology (Wuhan, China). Animals were maintained in accordance with the Guide for the Care and Use of Laboratory Animals published by the US National Institute of Health (NIH Publication No.85–23, revised 1996).

#### Transthoracic echocardiography

Prior to and 28 days following surgery, rats in the 28 days group were anesthetized (sodium pentobarbital, 50 mg/kg, i.p.) and lightly secured to a warming pad in the supine position, and their precordium was shaved. Transthoracic echocardiography was performed using a cardiac ultrasound machine with a 11.2 MHz transducer (Vivid 7; GE Healthcare, Fairfield, CT, USA). The heart was first imaged in the two-dimensional mode in the parasternal long-axis and parasternal short-axis views. Left ventricular (LV) areas were measured from the transverse sections and the LV short-axis lengths were used to calculate LV end-systolic diameters (LVESDs) or volumes (LVESVs) and end-diastolic diameters (LVEDDs) or volumes (LVEDVs) using the modified Simpson’s rule formula. The inner endocardial margin defined the LV lumen and the LVEF was derived using the following formula: LVEF = (LVEDV – LVESV)/LVEDV × 100. LV thickness was measured from the M-mode recording at the mid-papillary level. The results from three different cardiac cycles were averaged.

#### Hemodynamic measurements

Hemodynamic measurements were performed in the 3 h group. LV systolic pressure (LVSP) and end-diastolic pressure (LVEDP), the maximum rate of increase of LV systolic pressure (dp/dtmax) and heart rate (HR) were measured using a 1% heparinized short segment of a saline-filled PE 50 catheter connected to a solid-state miniature pressure transducer and the Philips apparatus (Integris Allura 12; Philips Healthcare, Heide, The Netherlands) (23).

#### Serum c-troponin I (c-TnI) measurement

Prior to sacrifice, blood (1.0 ml) was obtained from the femoral vein of each rat in the 24 h group at 6 and 24 h postoperatively. c-TnI was measured by immunofluorescence methods and an immunofluorescence assay (OPUS, Dade Behring, Inc., Mariani Cupertino, CA, USA).

#### Determination of von Willebrand factor (vWF) and endothelin-1 (ET-1)

Blood (2.0 ml) was obtained from the femoral vein of each rat in all 4 groups (3 h, 24 h, 7 days and 24 days) prior to sacrifice. Blood was centrifuged at 2,000 x g and the serum vWF and ET-1 levels were determined using antibodies in an antigen-based sandwich ELISA assay (von Willebrand Factor Elisa kit, Helena Laboratories, Beaumont, TX, USA; Endothelin-1 Quantikine ELISA kit, ELISAs More Quality Reagents from R&D Systems, Minneapolis, MN, USA).

#### Evaluating areas of no-flow

Three hours postinjection, a single bolus of thioflavin-S (1 ml/kg of 4%; Sigma, St. Louis, MO, USA) was injected via the jugular vein to stain the vascular endothelium *in vivo* (24). One min later, the heart was stopped in the diastolic phase by an intravenous injection of potassium chloride (1 ml, 10%) and removed immediately. The atrium and right ventricle were separated from the left ventricle. The left ventricle was then placed into a −20°C freezer for 5 min and sectioned into 10 or 11 cross-sectional slices (1 mm in thickness) along the long axis using a cutting apparatus (JP40; Shanghai Shiyuan Scientific Equipment Co., Ltd., Shanghai, China). Even slices (2,4,6,8 and 10) were fixed in formalin solution and used for light microscopic and immunohistochemical analyses. Uneven slices (1,3,5,7,9 and 11) were irradiated with ultraviolet light (wave length, 365 nm) to determine the no-flow zone (NF, area not perfused by thioflavin-S). The NF was traced and analyzed using NIH imaging software (http://rsb.info.nih.gov/nih-image/). The ratios of NF/LV area (NF/LV) from all examined slices were calculated and averaged.

#### Light microscopic analysis

LV tissue samples from the 3 h group were fixed in 10% buffered formalin solution for 24 h, embedded in paraffin, cut into 4-*μ*m sections and stained with hematoxylin and eosin (H&E), Carstair’s (25) and hematoxylin basic fuchsin picric acid (HBFP) (26), respectively. An Olympus-BX41TF microscope (Olympus, Tokyo, Japan) incorporated with Image-Pro Plus 4 (Media Cybernetics, Inc., Rockville, MD, USA) was employed to observe the micro-thrombosis in the coronary arteriole in H&E-stained slices. One hundred coronary arterioles with a diameter <100 *μ*m were randomly observed under a microscope (magnification, ×200) and the percentage of microthrombosis in these arterioles was determined. For animals studied 28 days postoperatively, hearts were embedded in paraffin, sectioned at 5-*μ*m intervals and stained with Masson’s trichrome (27). Myocardial leukocyte infiltration was observed in rats 24 h and 7 days postoperatively in H&E-stained sections. Leukocytes were counted in 10 randomly selected visual fields in each section and five sections per rat were examined.

#### Immunohistochemical analysis

LV tissue samples from the 3 h group were fixed in 10% buffered formalin solution for 24 h, embedded in paraffin and cut into 4-*μ*m sections for immunohistochemical analysis. Tissue sections were blocked with 10% normal serum for 1 h at 27°C. The blocker was removed and the primary antibody, vascular smooth muscle α-actin (VSMA-α) antibody (mouse monoclonal anti-VSMA; Santa Cruz Biotechnology Inc., Santa Cruz, CA, USA) was added. The tissue sections were incubated for 15 h at 4°C and washed with PBS, then the peroxidase block was performed with 0.5% H_2_O_2_ in methanol for 30 min. The respective biotinylated secondary antibody (rabbit anti mouse IgG, Santa Cruz Biotechnology Inc.) was added and incubated for 1 h at 27°C. After washing with PBS, the streptavidin peroxidase label (Zymed, San Diego, CA, USA) was added and incubated for 10 min at 27°C. The sections were washed with PBS and incubated with AEC color development substrate (Zymed) for a further 10 min at 27°C. Sections were washed in water and counterstained with Mayer’s hematoxylin. The vascular smooth muscle was dyed claybank and the arterioles (10–50 *μ*m) were counted in 10 random fields of vision in each section, and five sections per rat were examined under a light microscope (magnification, ×200). Arteriolar density (AD) was calculated using the following formula: AD = total arteriole number/10 x 5/ the area of one vision (mm^2^).

#### Western blot analysis

At 24 h, 7 days and 28 days after injection, the apex of the left ventricle was used for western blot analysis. Tissues were homogenized in PBS and centrifuged at 10,000 × g for 10 min at 4°C, then 70 *μ*g supernatant was lysed in electrophoresis buffer, boiled for 10 min and subsequently subjected to electrophoresis on a SDS-polyacrylamide gel. The separated blots were transferred to nitrocellulose membranes and blocked for 1 h in TTBS buffer containing 5% nonfat milk. The membranes were incubated overnight with primary anti-TNF-α or IL-6 polyclonal antibodies (TNF-α, 1:500 dilution; IL-6, 1:200 dilution) and then with horseradish peroxidase (HRP)-conjugated rabbit anti-goat IgG antibody (1:1,000 dilution) for 2 h at 37°C. Blots were detected by chemiluminescence and relative protein expression was quantified by scanning densitometry.

#### Statistical analyses

The data were analyzed using SPSS 13.0 (SPSS, Inc., Chicago, IL, USA). Data are presented as the mean ± SD. Comparisons among groups and different experimental stages were first carried out using a test of homogeneity of variances and then by two-way ANOVA, Tukey’s test (homogeneity of variance) and the Games-Howell test (heterogeneity of variance). A P-value of 0.05 was considered to indicate a statistically significant difference.

## Results

### 

#### Pilot study

Prior to the main study, a pilot study was performed to observe the blood pressure and heart rate changes induced by various concentrations of diltiazem. In our previous study (9), thrombosis was observed at 1 h, peaked at 3 h and decreased at 12 h, while heart rate decreased significantly at 1 min and returned to baseline level at 5 min after the injection of automicrothrombotic particulates. Therefore, diltiazem was infused at doses of 10, 50, 100 or 200 *μ*g/min/kg (n=8 for each dose) through the tail vein for 175 min with an infuser at 5 min after the automicrothrombotic particulate injection. The 200 *μ*g/min/kg dose of diltiazem significantly reduced blood pressure (<90 mmHg) and heart rate (<200 beats per minute), while the 100 *μ*g/min/kg dose did not affect the blood pressure but significantly reduced the heart rate (<250 beats per minute). Blood pressure and heart rate were not significantly affected by the 10 and 50 *μ*g/min/kg doses of diltiazem, and the beneficial effects of reducing the NF/LV area were greater using 50 *μ*g/min/kg diltiazem compared with 10 *μ*m/min/kg diltiazem (5% vs. 2%). As a result, diltiazem at a dose of 50 *μ*g/min/kg was selected for the main study.

#### Mortality

In the sham group, 2 rats died during the perioperative period (1 due to excess anesthesia, 1 due to massive hemorrhage). The remaining 38 rats were examined at 3 h (n=9), 24 h (n=10), 7 days (n=10) and 28 days (n=9). In the CME group, 3 animals died at 10 h, 26 h and 4 days postinjection, respectively. This was due to cardiac failure based on the postmortem pathological findings (pulmonary venous pleonaemia and pleural effusion). The remaining 35 rats were examined at 3 h (n=9), 24 h (n=9), 7 days (n=9) and 28 days (n=8). In the CME+DIL group, no rats died and 38 rats were examined at 3 h (n=10), 24 h (n=9), 7 days (n=10) and 28 days (n=9).

#### Heart rate changes

**Figure 1.**
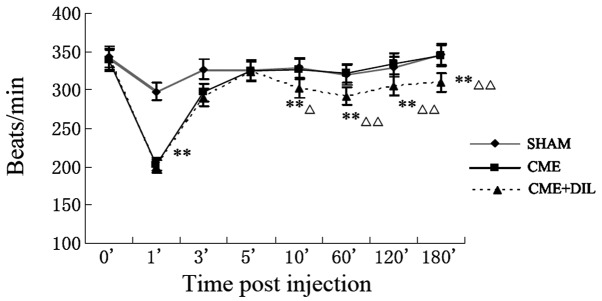
Heart rate changes postinjection. Following the injection of auto-microthrombotic particulates, the heart rate decreased significantly at 1 min and returned to baseline after 5 min in the CME and CME+DIL groups. The heart rate was significantly lower in the CME+DIL group than in the CME group. Data are presented as the mean ± SD. ^**^P<0.01 vs. the SHAM group; ^Δ^P<0.05, ^ΔΔ^P<0.01 vs. the CME group. CME, coronary thrombotic microembolism; DIL, diltiazem.

Following the injection of automicrothrombotic particulates, the heart rate decreased significantly at 1 min and returned to baseline level 5 min postinjection in the CME and CME+DIL groups, while it remained unchanged in the sham group (Fig. 1).

#### Transthoracic echocardiography

Preoperative values were comparable between the CME and CME+DIL groups. Four weeks postoperatively, the LVEDV was significantly lower, while the FS and LVEF were significantly higher, in the CME+DIL group compared with the CME group (Table I).

#### Hemodynamic measurements

In the 3 h group, dp/dtmax and-dp/dtmax were significantly higher, while LVEDP was significantly lower, in the CME+DIL group than in the CME group (Table II).

#### Levels of c-TNI at 6 and 24 h postinjection

**Figure 2.**
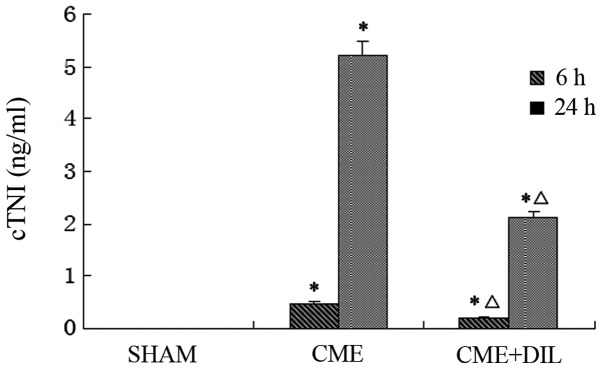
Serum c-troponin I (c-TNI) levels postinjection. c-TNI levels at 6 and 24 h after injection were significantly lower in the CME+DIL group than in the CME group. Data are presented as the mean ± SD. ^*^P<0.01 vs. the sham group; ^Δ^P<0.01 vs. the CME group. CME, coronary thrombotic microembolism; DIL, diltiazem.

c-TNI levels were significantly reduced in the CME+DIL group compared with those in the CME group at 6 and 24 h post-automicrothrombotic particulate injection (Fig. 2).

#### Levels of plasma vWF and ET-1 postinjection

Plasma vWF is regarded as a good indicator of endothelial dysfunction and has been shown to contribute to the activation of the coagulation cascade (28,29). Plasma vWF levels were significantly lower at 3 h following the injection of post-automicrothrombotic particulates in the CME+DIL group than in the CME group (Table III). Levels of ET-1, the endothelium-derived vasoconstrictor peptide, increase in response to myocardial ischemia and infarction (30,31). Plasma ET-1 levels at 3 h, 24 h and 7 days after the injection of post-automicrothrombotic particulates were also significantly lower in the CME+DIL group than in the CME group (Table III).

#### NF evaluation at 3 h postinjection

**Figure 3.**
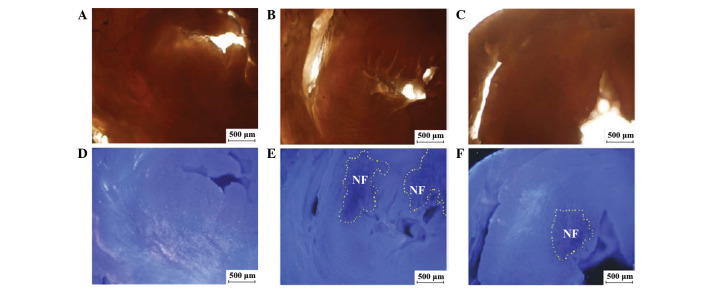
No-flow zone (NF) evaluation at 3 h postinjection. (A–C) Optical microscope images used as background references for the sham, CME and CME+DIL groups, respectively. Under ultraviolet light, the NF was identified as the region deficient in fluorescence of thioflavin-S in the (D) sham, (E) CME and (F) CME+DIL groups (magnification, ×40). The yellow dotted line delineates the NF. CME, coronary thrombotic microembolism; DIL, diltiazem.

The NF was evaluated by thioflavin S (blue fluorescence represented the perfused zone and non-fluorescent areas represented the NF when examined under ultraviolet light at a 365-nm wavelength). The NF/LV ratio was significantly lower in the CME+DIL group than in the CME group (5.6±2.5 vs. 11.2±2.7%, P<0.01; Fig. 3).

### Light microscope analyses

#### H&E staining at 3 h postinjection

**Figure 4.**
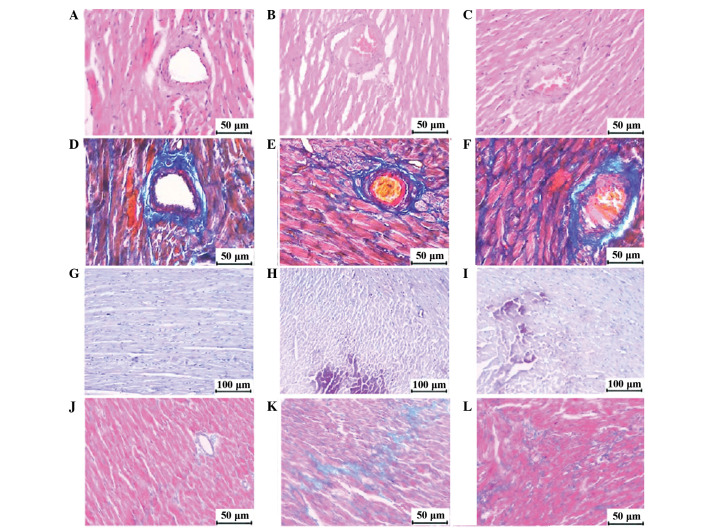
Light microscopic analyses. In H&E-stained slices (magnification, ×200), red thrombosis was not observed in arterioles of (A) the sham group, but was observed in the (B) CME and (C) CME+DIL groups. (D–F) In the sham, CME and CME+DIL groups, respectively, Carstair’s staining (magnification, ×200) showed that the major components of thrombosis in the CME group were fibrins (bright red), platelets (gray-blue to navy blue) and red cells (yellow). (G-I) In the sham, CME and CME+DIL groups, respectively, HBFP staining (magnification, ×100) showed cardinal red regions 3 h postinjection in the CME and CME+DIL groups. (J–L) In the sham, CME and CME+DIL groups, respectively, Masson staining at 4 weeks postinjection (magnification, ×200) showed increased collagen in the CME group and decreased collagen deposition in the CME+DIL group. H&E, hematoxylin and eosin; CME, coronary thrombotic microembolism; DIL, diltiazem; HBFP, hematoxylin basic fuchsin picric acid.

Three hours postinjection, red thrombi were identified in 18.2±4.5% of coronary arterioles with diameters <100 *μ*m in the CME group, compared with 10.4±2.5% in the CME+DIL group (P<0.01; Fig. 4B and C).

#### Carstair’s staining at 3 h postinjection

Different colors in Carstair’s staining represented different components of the thrombosis in arterioles; bright red for fibrin, gray-blue to navy blue for platelets, bright blue for collagen, red for muscle and clear yellow for red blood cells. Three hours postinjection, evidence of thrombosis was observed in the CME and CME+DIL groups. The major components of thrombosis were fibrins and platelets, and there was also red cell accumulation in the vascular lumen (Fig. 4E and F).

#### HBFP staining at 3 h post injection

HBFP staining was used to detect early myocardial ischemia or infarct regions. The normal myocardium was stained yellow or yellow-brown, and the ischemic or necrotic myocardial tissue was stained cardinal red. The ischemic area (IA) was calculated using the following formula: IA (%) = IA/area of field of vision x 100. The IA was 6.3±1.2% in the CME group and reduced to 3.3±1.2% in the CME+DIL group (P<0.01; Fig. 4H and I).

#### Masson staining 28 days postoperatively

Masson staining was carried out in the 28 days postinjection group. Cardiomyocytes were stained red and collagen stained blue. The collagen volume fraction (CVF = area of collagen/area of the field of vision × 100%) was measured. The CVF was significantly lower in the CME+DIL group (Fig. 4L) than in the CME group (Fig. 4K; 5.38±1.46 vs. 2.60±1.07%, P<0.01).

#### Inflammatory cell infiltration

**Figure 5.**
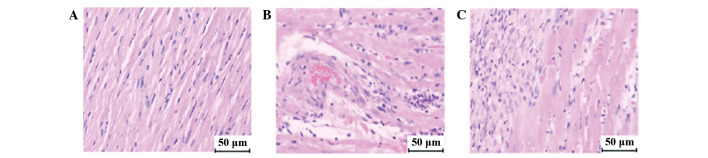
Inflammatory cell infiltration by hematoxylin and eosin (H&E) staining. (A) The sham group. (B) Increased polymorphonuclear leukocyte infiltration was observed in the microinfarct zone in the CME group at 24 h postinjection. (C) Increased macrophage and leukomonocyte infiltration was observed near the microinfarct zone in the CME+DIL group at 7 days postinjection (magnification, ×200). CME, coronary thrombotic microembolism; DIL, diltiazem.

At 24 h postinjection, coagulative necrosis occurred in the microinfarct zone and polymorphonuclear leukocyte infiltration was observed around the blocked vessel in the CME group (Fig. 5B). Seven days postinjection, the majority of infiltrated leukocytes were macrophages and leukomonocytes in the CME+DIL group (Fig. 5C). The leukocyte counts were significantly lower in the CME+DIL group than in the CME group 24 h and 7 days postoperatively (Table IV).

#### Immunohistochemical staining

**Figure 6.**
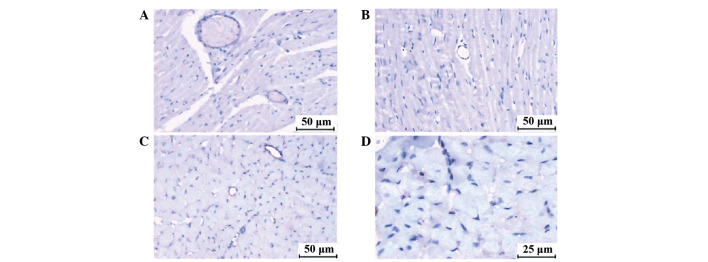
Immunohistochemical staining. Positive expression of vascular smooth muscle α-actin in vascular smooth muscle cells (magnification, ×200). Two arterioles of different diameters were observed in the (A) CME and (C) CME+DIL groups (magnification, ×200). An example of (B) a venule (magnification, ×200) and (D) a capillary (magnification, ×400) in the CME+DIL group. CME, coronary thrombotic microembolism; DIL, diltiazem.

At 3 h postinjection, VSMA-α was expressed in vascular smooth muscle cells and the vascular smooth muscle was dyed claybank in order to count the arterioles (10–100 *μ*m). Fig. 6 shows arterioles with different diameters in the CME and CME+DIL groups. Immunohistochemical staining analysis indicated that the number of arterioles with a diameter in the range of 10–50 *μ*m, particularly arterioles with diameters of 20–50 *μ*m, was significantly higher in the CME+DIL group than in the CME group at 3 h postinjection (Table V).

#### Western blot analysis

**Figure 7.**
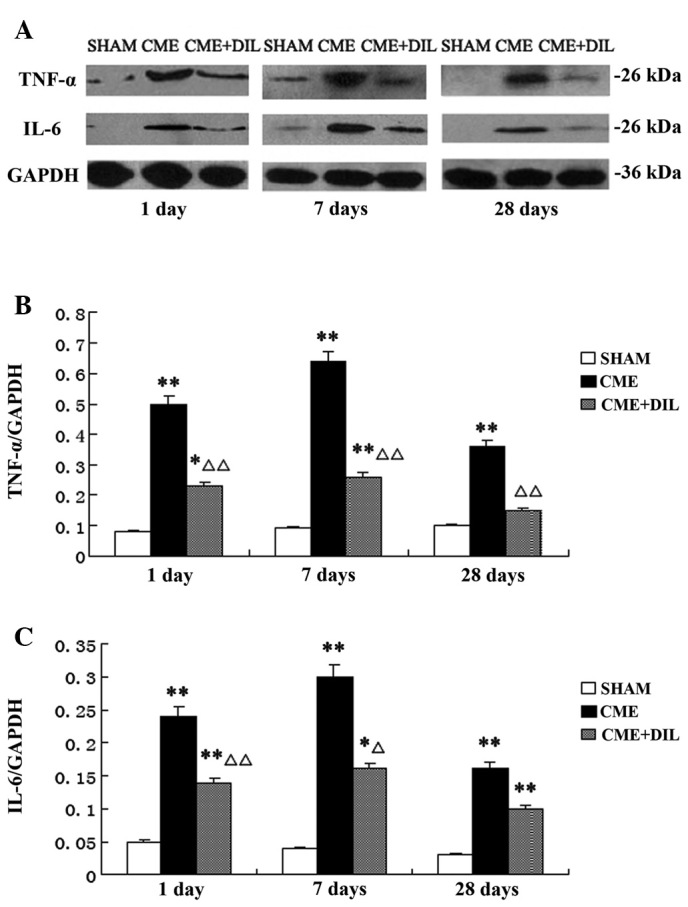
Western blot analysis of TNF-α and IL-6. (A) Expression levels of TNF-α and IL-6 at different time-points. (B) Representation of TNF-α/GAPDH at different time-points. (C) Representation of IL-6/GAPDH at different time-points. Data are presented as the mean ± SD. ^*^P<0.05, ^**^P<0.01 vs. the sham group. ^Δ^P<0.05, ^ΔΔ^P<0.01 vs. the CME group. TNF-α, tumor necrosis factor α; IL-6, interleukin-6; CME, coronary thrombotic microembolism; DIL, diltiazem.

The myocardial protein expression levels of TNF-α and IL-6 were significantly downregulated in the CME+DIL group compared with those in the CME group at various time-points postinjection (Fig. 7).

## Discussion

The present study demonstrated that the injection of auto-microthrombotic particulates into the aorta of male Sprague Dawley rats successfully induced CME, arteriolar thrombosis, histologically-confirmed ischemic regions and NF. This confirmed the pathological changes that were shown in this model in a previous study (9).

Results from this model demonstrated that the components of the thrombosis in coronary arterioles were fibrin, aggregated platelets and red blood cells, indicating the presence of vessels that were obstructed by automicrothrombotic particulates and newly formed thrombosis *in situ*. Increased vWF and ET-1 levels, indicators of endothelial function (32), at 3 h postinjection in CME rats indicated that microthrombotic particulates may also induce microvascular endothelial injury. Acute myocardial injury was demonstrated by increased c-TnI levels at 6 and 24 h and myocardial infarctlets in HBFP-stained myocardium at 3 h post-automicrothrombotic particulate injection in this model. Immunohistochemical staining analysis indicated that the number of 10–50 *μ*m diameter arterioles (particularly 20–50 *μ*m) was significantly reduced in the CME group at 3 h postinjection compared with the number in the sham rats. Therefore, the injection of automicrothrombotic particulates induced not only arteriolar thrombosis, but also arteriolar spasm. Moreover, increased serum c-TnI levels and myocardial leukocyte infiltration at early time-points postinjection and prolonged inflammatory responses indicated by increased myocardial TNF-α and IL 6 expression resembled typical inflammatory responses post-ischemia (33). This finding is in line with previous reports showing that an inflammatory reaction was the most significant mechanism resulting in systolic heart failure in CME and that the inflammatory mediator TNF-α may be causal in contractile dysfunction following CME (34–38). The interactions between microembolism, plaque fissuring, arrhythmia and dysfunction have been summarized previously (5,9,12). Thus, there may be a correlation between microembolism and the NF phenomenon in that increased myocardial collagen content demonstrated by histology and reduced cardiac function shown by transthoracic echocardiography at 4 weeks may be the sequential changes induced by microembolism. Briefly, coronary microembolism/ coronarymicrothrombosis may result in endothelial damage/ dysfunction as well as arteriolar spasm, leading to coronary vascular resistance increase, no reflow, microinfarctlets inflammatory reaction, myocardial remodeling and cardiac dysfunction.

The aim of this study was to explore the feasibility of using the present rat CME model to reflect the therapeutic effects of clinically effective medication on CME injury. The effects of intravenous diltiazem were therefore evaluated in this animal model. Diltiazem is a calcium channel antagonist that inhibits myocardial calcium entry by blockade of voltage-dependent membrane calcium channels. Although calcium channel antagonists all inhibit calcium channel conductance, they exhibit considerable selectivity of action in terms of vasodilation, negative inotropic and chronotropic effects (37,39). Previous clinical studies have shown that calcium antagonists may attenuate microvascular spasm by relaxing small vascular smooth muscle (7,8,40), and intravascular application of diltiazem may attenuate coronary artery spasm in patients with microvascular angina (19,20). In the current study, it was demonstrated that intravenous diltiazem (1 mg/ml, 50 *μ*g/min/kg) administered at 5 min post-automicrothrombotic particulate injection for 175 min improved cardiac function, attenuated the reduction in the number of arterioles (diameter 10–50 *μ*m) and reduced the NF in the present CME model, possibly through attenuating microvascular spasm. Moreover, diltiazem also reduced myocardial ischemia, endothelia dysfunction and inflammatory responses, as indicated by changes in c-TnI, plasma vWF and ET-1 levels, as well as the myocardial protein expression levels of TNF-α and IL-6. It was also demonstrated that the number of red thrombi reduced immediately after diltiazem application in H&E-stained myocardial tissue, the ischemic area in HBFP-stained myocardial samples was reduced 3 h postinjection and the CVF in Masson-stained myocardial samples was reduced 28 days postinjection. These results are thus in line with previous findings showing that treatment of the ischemic myocardium with calcium channel blockers attenuates ultrastructural myocardial injury (41,42), decreases calcium influx (42,43) and improves postischemic left ventricular segmental function (43–48). The NF area was reduced but did not disappear completely in the CME+DIL group. This may indicate that part of the capillary network was already undergoing necrotic changes and was unable to be recovered following diltiazem treatment. However, diltiazem did improve automicrothrombotic particulate-induced functional no-reflow by reducing myocardial spasm (47).

Large animal CME models have been widely used in microembolism research (49,50). The rat CME model used in the present study differs from the large animal CME models in the following respects: i) This model mimicked *in vivo* arteriole blockade by various sizes of microthrombi following atherosclerotic plaque rupture; ii) the components of auto-microthrombotic particulates are similar to thrombi *in vivo*, including fibrin, platelets and blood corpuscle; iii) automicrothrombotic particulates are easy to obtain and do not require elaborate equipment in the laboratory; iv) rat models are more economical compared with large animal models; and v) rat hearts are smaller and the entire heart may be easily sampled with few histological sections.

Notably, it is difficult to define the exact mechanism of action for diltiazem and to differentiate the anti-vasospasm and -vasoconstriction effects of diltiazem with the data available for this model. Therefore, further studies are required to explore these points.

In conclusion, this animal model mimicked certain pathological changes induced by coronary embolization that have been observed in clinical patients with acute coronary syndromes and in patients who have undergone revascularization procedures (fibrinolytics or transcatheter recanalization during surgical or percutaneous procedures, or prior embolization before procedures). Intravenous diatiazem reduced automicrothrombotic particulate injection-induced myocardial injury. Thus, this model may be used to test the effects of drugs that have the potential to attenuate CME and arteriolar thrombosis-induced myocardial injury.

## Figures and Tables

**Table I.** Two-dimensional mode transthoracic echocardiography results.

Variable	Status	Sham group	CME group	CME+DIL group
LVEDD (mm)	Pre-op	5.66±0.59 (n=10)	5.84±0.47 (n=10)	5.88±0.39 (n=10)
	Post-op	5.77±0.42 (n=8)	6.65±0.61^a,b^ (n=8)	6.20±0.44 (n=9)
LVESD (mm)	Pre-op	2.88±0.24 (n=10)	2.95±0.21 (n=10)	2.90±0.19 (n=10)
	Post-op	2.92±0.13 (n=8)	4.58±0.45^a,b^ (n=8)	3.73±0.43^a–c^ (n=9)
LVEDV (ml)	Pre-op	0.47±0.11 (n=10)	0.48±0.10 (n=10)	0.49±0.09 (n=10)
	Post-op	0.47±0.05 (n=8)	0.92±0.10^a,b^ (n=8)	0.71±0.10^a–c^ (n=9)
LVESV(ml)	Pre-op	0.09±0.02 (n=10)	0.09±0.01 (n=10)	0.09±0.01 (n=10)
	Post-op	0.09±0.02 (n=8)	0.38±0.04^a,b^ (n=8)	0.25±0.05^a–c^ (n=9)
FS (%)	Pre-op	48.9±1.7 (n=10)	49.4±1.2 (n=10)	50.7±2.7 (n=10)
	Post-op	49.3±1.9 (n=8)	31.2±2.1^a,b^ (n=8)	40.4±4.3^a–c^ (n=9)
LVEF (%)	Pre-op	81.3±1.6 (n=10)	80.3±2.4 (n=10)	80.9±2.6 (n=10)
	Post-op	80.0±3.7 (n=8)	58.2±6.8^a,b^ (n=8)	64.5±9.8^a,b^ (n=9)

Data are presented as the mean ± SD.

a P<0.01 vs. preoperative in the same group;

b P<0.01 vs. postoperative in the sham group;

c P<0.01 vs. postoperative in the CME group.

LVEDD, left ventricular end-diastolic dimension; LVESD, left ventricular end-systolic dimension; LVESV, left ventricular end-systolic volume; LVEDV, left ventricular end-diastolic volume; FS, fractional shortening; LVEF, left ventricular ejection fraction; CME, coronary thrombotic microembolism; DIL, diltiazem.

**Table II.** Hemodynamic measurements.

Group	n	LVSP (mmHg)	LVEDP (mmHg)	dp/dtmax (mmHg/s)	–dp/dtmax (mmHg/s)
Sham	9	150.1±9.8	5.4±2.9	5605±693	−5034±394
CME	9	104.3±9.6^a^	15.3±2.8^a^	2986±236^a^	−2612±239^a^
CME+DIL	10	106.0±7.3^a^	10.8±2.0^a,c^	3407±359^a,b^	−2905±316^a^

Data are presented as the mean ± SD.

a P<0.01 vs. the sham group;

b P<0.05,

c P<0.01 vs. the CME group.

LVSP, left ventricular systolic pressure; LVEDP, left ventricular end-diastolic pressure; dp/dtmax, the maximum rate of increase of the LV systolic pressure; -dp/dtmax, the maximum rate of decrease of the LV systolic pressure; CME, coronary thrombotic microembolism; DIL, diltiazem.

**Table III.** Plasma vWF and ET-1 levels postinjection.

Group	Variable	Time postinjection			

		3 h	24 h	7 days	28 days
Sham	vWF (ng/ml)	6.08±0.29 (n=9)	5.98±0.25 (n=9)	5.94±0.26 (n=9)	6.02±0.29 (n=8)
	ET-1 (pg/ml)	50.2±0.24 (n=9)	52.2±0.26 (n=9)	51.2±0.23 (n=9)	52.2±0.29 (n=8)
CME	vWF (ng/ml)	7.80±0.58^a^ (n=9)	6.61±0.46^a^ (n=10)	6.10±0.41 (n=10)	6.23±0.35 (n=9)
	ET-1 (pg/ml)	93.6±1.24^a^ (n=9)	154.2±2.46^a^ (n=10)	114.8±2.46^a^ (n=10)	84.0±1.35^a^ (n=9)
CME+DIL	vWF (ng/ml)	6.95±0.59^a,b^ (n=10)	6.31±0.49 (n=9)	6.06±0.36 (n=10)	6.13±0.39 (n=9)
	ET-1 (pg/ml)	73.2±0.48^a,b^ (n=10)	100.2±0.49^a,b^ (n=9)	78.6±0.76^a,b^ (n=10)	74.8±1.68^a^ (n=9)

Data are presented as the mean ± SD.

a P<0.01 vs. the sham group;

b P<0.01 vs. the CME group.

vWF, von Willebrand factor; ET-1, endothelin-1; CME, coronary thrombotic microembolism; DIL, diltiazem.

**Table IV.** Leukocyte infiltration postinjection (leukocytes/mm^2^).

Group	Time postinjection		

	24 h	7 days	28 days
Sham	158±42 (n=9)	160±42 (n=9)	157±22 (n=8)
CME	930±126^a^ (n=10)	836±105^a^ (n=10)	160±24 (n=9)
CME+DIL	652±112^a,b^ (n=9)	322±66^a,b^ (n=10)	159±22 (n=9)

Data are presented as the mean ± SD.

a P<0.01 vs. sham group;

b P<0.01 vs. CME group.

CME, coronary thrombotic microembolism; DIL, diltiazem.

**Table V.** Number of arterioles at 3 h postinjection (s/mm^2^).

Arteriole diameter	Group		

	Sham	CME	CME+DIL
10–20 *μ*m	2.61±0.18 (n=9)	2.15±0.26^b^ (n=9)	2.4±0.19^c^ (n=10)
20–50 *μ*m	0.70±0.06 (n=9)	0.32±0.10^b^ (n=9)	0.59±0.14^d^ (n=10)
50–100 *μ*m	0.30±0.03 (n=9)	0.28±0.05 (n=9)	0.32±0.04 (n=10)

Data are presented as the mean ± SD.

a P<0.05,

b P<0.01 vs. the sham group;

c P<0.05,

d P<0.01 vs. the CME group.

CME, coronary thrombotic microembolism; DIL, diltiazem.
